# The Association between Tear Film Thickness as Measured with OCT and Symptoms and Signs of Dry Eye Disease: A Pooled Analysis of 6 Clinical Trials

**DOI:** 10.3390/jcm9113791

**Published:** 2020-11-23

**Authors:** Gerhard Garhöfer, Valentin Aranha Dos Santos, Hannes Stegmann, Doreen Schmidl, Narine Adzhemian, René M. Werkmeister, Leopold Schmetterer

**Affiliations:** 1 Department of Clinical Pharmacology, Medical University of Vienna, 1090 Vienna, Austria; gerhard.garhoefer@meduniwien.ac.at (G.G.); doreen.schmidl@meduniwien.ac.at (D.S.); adzhemian@gmail.com (N.A.); rene.werkmeister@meduniwien.ac.at (R.M.W.); 2 Center for Medical Physics and Biomedical Engineering, Medical University of Vienna, 1090 Vienna, Austria; valentinaranha1@gmail.com (V.A.D.S.); hannes.stegmann@meduniwien.ac.at (H.S.); 3 Christian Doppler Laboratory of Ocular and Dermal Effects of Thiomers, Medical University of Vienna, 1090 Vienna, Austria; 4 Helmholtz Moscow Research Institute of Eye Diseases, 105062 Moscow, Russia; 5 Singapore Eye Research Institute, Singapore National Eye Centre, Singapore 169856, Singapore; 6 Lee Kong Chian School of Medicine, Nanyang Technological University, 59 Nanyang Drive, Singapore 308232, Singapore; 7 Academic Clinical Program, Duke-NUS Medical School, Singapore 169857, Singapore

**Keywords:** dry eye disease, optical coherence tomography, tear film thickness, signs and symptoms, ocular surface disease

## Abstract

Purpose: To determine the association between tear film thickness (TFT) as measured with ultra-high resolution optical coherence tomography (UHR-OCT) and signs and symptoms of dry eye disease (DED). Methods: A total of 450 eyes from 225 patients with DED from six different randomized clinical trials were included in this pooled analysis. In all subjects, TFT was measured with a custom-built UHR-OCT system. Symptoms of DED were quantified using a standardized Ocular Surface Disease Index (OSD)I questionnaire and clinical signs including tear film break up time (TFBUT) and Schirmer I test were assessed. Associations of the average TFT with OSDI, TFBUT, and Schirmer I test were calculated using a linear regression analysis. Results: The average TFT of the included sample (mean age, 45.0 ± 13.3 years; 65% female) was 4.2 ± 0.5 µm and the OSDI 36.2 ± 10.4. A significant negative correlation was found between TFT and OSDI (r = −0.36 to −0.31; *p* < 0.001). Tear break up time and Schirmer I test were not correlated with OSDI. Significant albeit weak correlations were found between TFT and TFBUT (r = 0.17 to 0.25; *p* < 0.01) as well as Schirmer I (r = 0.36 to 0.37; *p* < 0.001). Subgroup analysis revealed that the correlation was stronger in the subjects with abnormal Schirmer I (<15 mm; r = 0.50 to 0.54; *p* < 0.001). Conclusions: The present study demonstrates an objective measurement of TFT using a novel OCT approach for DED that correlates with symptoms and signs of DED. Our data are consistent with the idea that TFT represents the aqueous-deficient component of DED.

## 1. Introduction

Dry eye disease (DED) is a highly prevalent ocular condition that is characterized by alterations in the homoeostasis of the precorneal tear film and the ocular surface [1,2,3,4]. Although the understanding of DED as a multifactorial disease has constantly increased in the past years, diagnosis and follow up are still challenging. Studies in patients with DED have consistently reported weak associations between classical anatomical and functional markers such as corneal staining and the symptoms of the disease [5,6,7,8,9,10]. Thus, direct visualization and quantification of the most important component in DED, namely the tear film, are valuable for both diagnosis and follow up.

Given that the total thickness of the human tear film is thin, ranging between 3 and 5 micrometers over the central cornea, quantification of tear film thickness (TFT) and its components requires highly sophisticated technical approaches [11]. These include classical interferometry based methods, by which TFT measurements can be achieved via thickness-dependent fringes, angle-dependent fringes or wavelength-dependent fringes [12,13,14], thermal imaging [15], and others [16].

More recently, optical coherence tomography (OCT) has been proposed to study TFT and tear film dynamics in vivo [17,18,19]. However, up to now, commercial OCT systems do not provide sufficient axial resolution to visualize the precorneal tear film. We have recently introduced a custom-built ultra-high resolution OCT system that offers resolution in the order of 1 µm [20] due to a light source with a high bandwidth [21,22].

Using this system, we recently reported in a small cross-sectional study a correlation between TFT and symptoms of DED using the Ocular Surface Disease Index (OSDI) as well signs of DED such as tear film break-up-time (TFBUT) and tear osmolarity [23]. These preliminary data indicate that TFT may be a more accurate indicator of dry eye status than current clinical tests such as TFBUT and Schirmer I test.

The aim of the present study was to extend these findings and to examine the relation between TFT with symptoms and signs of DED in a larger study population. We hypothesize that TFT, an objective measurement will be more correlated with symptoms of DED than current clinical tests. We also hypothesized that inter-eye differences between different signs of DED may show a better correlation with TFT than inter-subject differences.

## 2. Material and Methods

### 2.1. Patients

The present analysis includes pooled data from subjects with DED from six randomized clinical trials performed at the Department of Clinical Pharmacology in Vienna. The study protocols were approved by the Ethics Committee of the Medical University of Vienna (EC project numbers: 1226/2012; 1814/2014; 1039/2013; 1064/2016; 1844/2016; 1839/2016) and were performed in adherence to the Declaration of Helsinki and the Good Clinical Practice (GCP) guidelines of the European Union. All subjects gave written informed consent before inclusion. In all these trials, the outcome was the effect of eye drops on TFT. Five of the studies have already been published [24,25,26,27,28], data from one study is yet unpublished. As these studies include different interventions, for the current analysis only baseline data from both eyes were included. In summary, in the present analysis, a total of 450 eyes from 225 patients were included. Data were included in the present analysis only when high-quality OCT scans were obtained from both eyes.

Main inclusion criteria were age over 18 years and signed and dated written informed consent. Patients had to have a history of the disease for at least 3 months, a TFBUT ≤10 s or Schirmer I test ≤10 mm/5 min and an OSDI score ≥13 points. Patients suffering from Sjögren’s syndrome or Stevens Johnson syndrome as well as patients not on stable concomitant medication that could have an influence on DED were excluded. Morever, intake of immune-suppressants was not allowed. All participating subjects did undergo a pre-study screening, which was scheduled in the 14 days before the baseline study day. This pre-study visit consisted of medical history, pregnancy test in women with childbearing potential, assessment of symptoms of DED using OSDI questionnaire, an ophthalmic examination including slit-lamp biomicroscopy and indirect funduscopy, visual acuity using the ETDRS acuity charts, measurement of TFBUT and Schirmer I test. All study participants had to abstain from administration of topical lubricants in the 24 h before the study day to avoid any undesirable influence on TFT.

### 2.2. Methods

#### 2.2.1. Ocular Surface Disease Index (OSDI)

The OSDI (Allergan plc, Irvine, CA, USA) is a well-accepted questionnaire to assess the symptoms of DED [29]. The OSDI questionnaire consists of 12 items that are graded on a scale of 0 to 4, where 0 indicates none of the time; 1, some of the time; 2, half of the time; 3, most of the time; and 4, all of the time. The total OSDI score is calculated as sum of scores for all questions answered ×100/total number of questions answered ×4. The minimum OSDI score is 0 and indicates no dry-eye related symptoms and the maximum score is 100 indicating maximum disability due to DED. Score values of < 13 are considered as the threshold for DED [22].

#### 2.2.2. Tear Film Thickness Using OCT

A custom-built OCT system was used for TFT measurements that we have described in detail previously [20]. As a light source, a Ti:sapphire laser (Integral OCT; Femtolasers Produktions GmbH, Vienna, Austria) with a bandwidth of 170 nm and a central wavelength of 800 nm was employed at a power of 600 µW on the corneal surface. This results in a theoretical axial resolution of 1.3 µm in the tissue. The lateral resolution of the system is given by the numerical aperture and is approximately 21 µm at the interface between tear film and corneal epithelium. Before the measurements, patients were asked to blink once and the measurement procedure started immediately after opening of the eyes. Recording consists of three 3-dimensional volumes with a size of 4 × 4 × 1 mm^3^ (horizontal × vertical × depth) containing 512 × 256 × 1024 voxels. The recording of each volume takes 1 s resulting in a total measurements time of 3 s. The central TFT was calculated as the mean of the 15 horizontal frames above the central specular reflex that occurs at the apex of the cornea. The first volume was not used for data analysis, because TFT immediately after blinking shows considerable variability (unpublished observation). As such only volumes 2 and 3 were analyzed and the mean value obtained 1–3 s after blinking was used to calculate TFT. A typical ultra-high resolution OCT image is shown in Figure S1.

#### 2.2.3. Tear Film Break up Time (TFBUT)

Tear film break up time (TFBUT) testing is a clinical test used to assess for evaporative dry eye disease. We followed the guidelines published in the Report of the International Dry Eye Work Shop [10]. For this purpose 5 µL sodium fluorescein drops (Minims-Fluorescein Sodium 2.0%, Chauvin Pharmaceuticals Ltd., London, UK) were instilled into the patient’s eye and the patient was instructed to blink naturally several times to distribute the fluorescein over the entire ocular surface. Within 10–30 s after instillation of the dye, the patient was asked to stop blinking and look straight. The tear film was observed during illumination with a broad beam of cobalt blue using a Wratten 12 yellow filter to enhance visibility with 10× magnification. The TFBUT was recorded as the number of seconds elapsing between the last blink and the appearance of the break up in the tear film. Values of TFBUT below 10 s are considered abnormal.

#### 2.2.4. Schirmer I Test

Schirmer I test without anesthesia was performed according to the protocol published in the Report of the International Dry Eye Work Shop [10] to evaluate tear production. The Schirmer paper strip (Schirmer-Plus, Gecis, Neung Sur Beuvron, France) was inserted in the eye over the lower lid margin, midway between the middle and outer third. The patient kept the eye closed afterwards for 5 min and the wetting of the Schirmer paper was evaluated. Abnormal aqueous tear production is supported by values <15 mm.

### 2.3. Data Analysis

For statistical analysis, both eyes were treated separately. Mean and standard deviation (SD) was calculated for each outcome parameter. The difference in TFT, TFBUT, and Schirmer was calculated between right and left eyes. The eyes were divided into those having normal TFBUT versus those having abnormal TFBUT (<10 s) as well as in those having normal Schirmer I versus those having abnormal Schirmer I (<15 mm). The association between the outcome variables was performed using linear regression analysis. All tests were performed at a significance level of *p* = 0.05. Statistical analysis was carried out using CSS Statistica for Windows^®^ (Statsoft Inc., Version 6.0, Tulsa, OK, USA).

## 3. Results

The mean age of the 225 patients with DED included in the present study was 45.0 ± 13.3 years with mean duration of DED of 6.3 ± 4.8 years. Majority of patients were female (*n* = 146; 65%). The severity of DED ranged from mild to moderate with an average OSDI of 36.2 ± 10.4. Table 1 summarizes the results for TFT, TFBUT, and Schirmer I stratified by right and left eyes. Out of the 450 study eyes 283 (63%) had abnormal TFBUT (<10 s) and 191 (42%) had abnormal Schirmer I test (<15 mm). Neither OSDI nor signs of DED correlated with patient’s age, duration of DED or gender (all r values below 0.11, data not shown).

**Figure 1:**
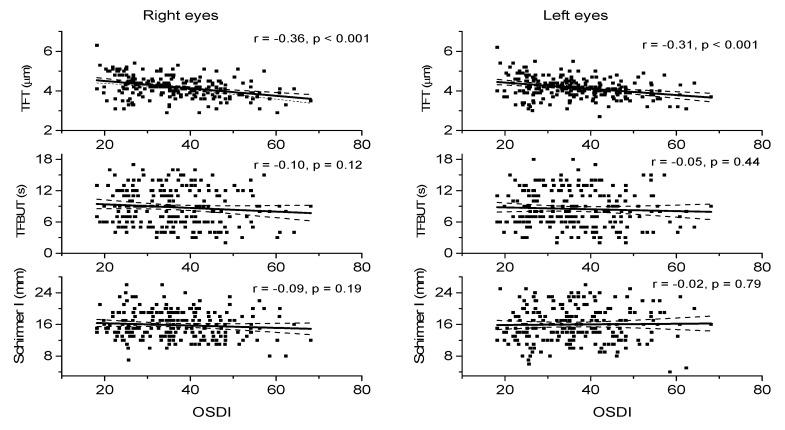
Correlation between Ocular Surface Disease Index (OSDI) and Tear Film Thickness (TFT), Tear Film Break Up Time (TFBUT) and Schirmer I test (Schirmer I). Data are separately presented for right and left eyes (*n* = 225). The regression line (solid line) and the 95% confidence interval are shown (dashed lines).

Regression analysis between symptoms and signs of DED are shown in Figure 1. A significant negative association was only found between OSDI and TFT (Figure 1, upper panel). No significant associations were seen between OSDI and TFBUT (Figure 1, middle panel) or Schirmer I (Figure 1, lower panel).

**Figure 2:**
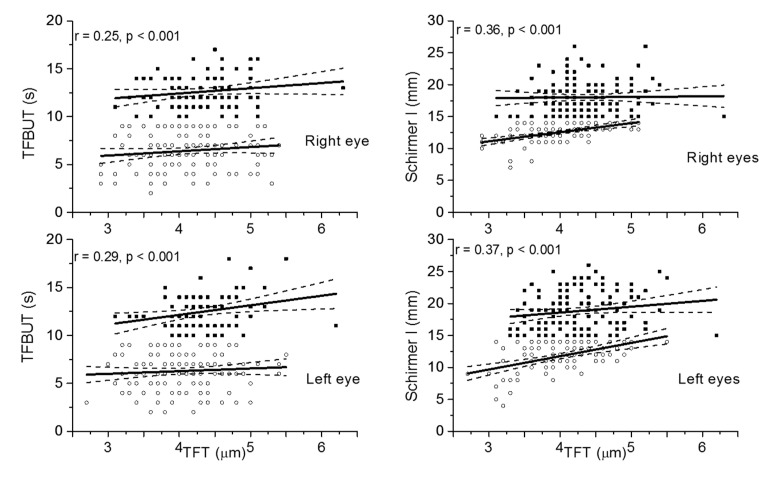
Correlation between Tear Film Thickness (TFT) and Tear Film Break Up Time (TFBUT) and Schirmer I test (Schirmer I). Correlations are presented for overall values, details can be found in Table 2). Data are separately presented for right and left eyes (*n* = 225). The data representing normal TFBUT (>9 s) and normal Schirmer I (>14 mm) are presented in black squares. The data representing abnormal TFBUT and abnormal Schirmer I are presented in open circles. The regression line (solid line) and the 95% confidence interval are shown (dashed lines).

The correlation between TFT and TFBUT as well as Schirmer I is presented in Figure 2. TFT was significantly correlated with TFBUT (Figure 2, left panels). TFT was also correlated with Schirmer I (Figure 2, right panels). No significant association was observed between Schirmer I and TFBUT for either right or left eyes (data not shown).

We then performed a subgroup analysis to examine the relation between TFT and TFBUT as well as Schirmer I by applying a TFBUT cutoff of <10 s and Schirmer I cutoff of <15 mm (Table 2). When TFBUT data were divided into normal (≥10 s) and abnormal (<10 s) values, no significant association was found for eyes with abnormal TFBUT. In eyes with normal TFBUT (*n* = 167), a significant association was found only in the left eyes and not in right eyes. The correlation line was steeper for the entire population than for the subgroups with either normal or abnormal TFBUT (Table 2).

When Schirmer I data were divided into normal (≥15 mm) and abnormal (<15 mm) values, a relatively strong correlation was found between TFT and Schirmer I for eyes with abnormal Schirmer I test. However, in eyes with normal Schirmer I, no significant correlation was found. The regression line was steepest for the entire study population (Table 2).

**Figure 3:**
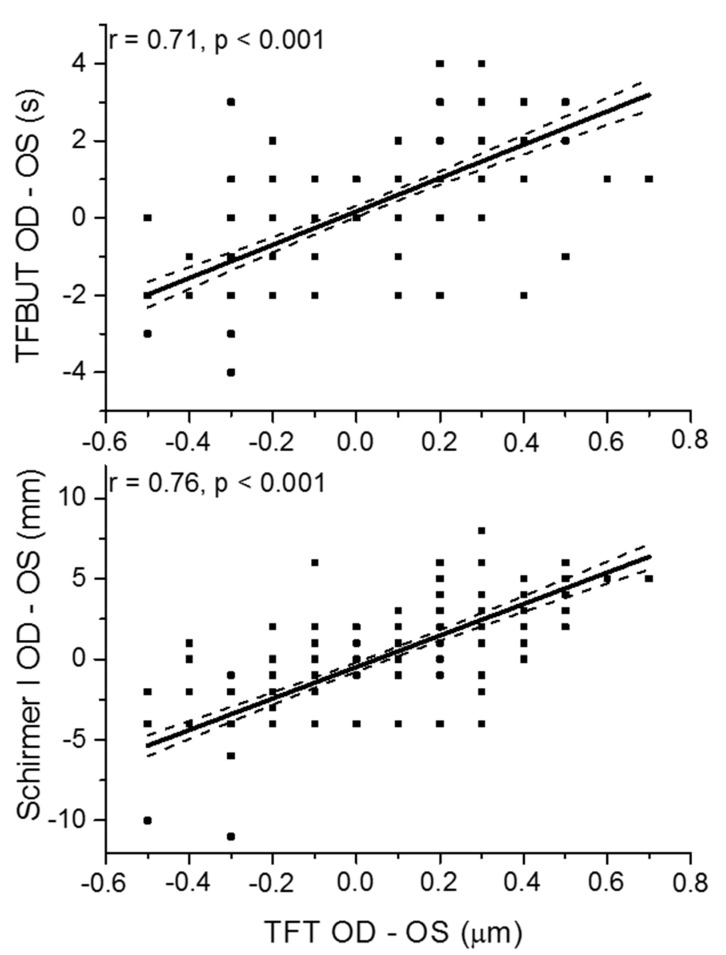
Correlation between inter-eye difference in Tear Film Thickness (TFT OD-OS) and inter-eye difference Tear Film Break Up Time (TFBUT OD-OS) and inter-eye difference Schirmer I test (Schirmer I OD-OS; *n* = 225). The regression line (solid red) and the 95% confidence interval are shown (dashed red).

Figure 3 shows the correlation between TFT and TFBUT as well as Schirmer I using inter-eye differences analysis (difference between right and left eyes). Compared to eye level analysis (Figure 2), the associations between inter-eye differences were stronger for both the correlation between TFT and TFBUT (Figure 3, upper panel) as well as between TFT and Schirmer I (Figure 3, lower panel) than at eye level analysis.

## 4. Discussion

The present study shows a significant, but relatively weak association between TFT and OSDI as well as TFT and TFBUT, which is in line with our previous results [23]. Our previous study was, however, much smaller and included patients with a wider range of OSDI. The present study extents our previous results and adds further to the understanding of the association between TFT and signs and symptoms of DED. In particular, we observed a weak correlation between TFT and Schirmer I in the entire study population, but a much stronger correlation when subjects with low Schirmer I were analyzed separately. This indicates that in patients who have low tear production, TFT is partially thin because of insufficient of tear production.

In addition, we observed a relatively strong association between inter-eye differences for TFT and TFBUT as well as for TFT and Schirmer. These results indicate that the weak correlation between these parameters in the overall population is to a large degree related to hitherto unidentified factors. Factors that could potentially explain the weak correlation are composition of tears and surface tension. Based on our data, one may hypothesize that the latter factors show relatively little inter-eye variability but high inter-individual variability, which may explain at least partially the results of the present study. Further experiments are, however, required to test this possibility.

In the present study, the association between clinical signs and reported symptoms of DED patients was weak, with TFT being the only variable significantly correlating with OSDI. This finding is in keeping with data from our own group as well as from previous other investigators [5,8,23,30] all equivocally reporting a lack or an insignificant association between the results of clinical tests for dry eye the ocular symptoms experienced by patients. Although the reason for this poor correlation is still unclear, it may be hypothesized that a stronger association exists mainly in patients with severe DED. This hypothesis is also supported by a study in patients with primary Sjögren syndrome that revealed correlations between Schirmer I test values and a variety of DED symptoms [31]. Of note, the DED in patients with Sjögren syndrome is characterized primarily to aqueous deficiency [32] due to insufficient production of tear fluid. The observation that TFT, but not TFBUT or Schirmer I were correlated with DED symptoms may also be related to the better reproducibility of the OCT-based approach [20,33,34,35].

Additionally, it needs to be considered that different clinical signs of DED also reflect different phenotypes of DED [8]. Obviously, low TFT and Schirmer test may rather be indicative of aqueous deficient DED, whereas low TFBUT may rather reflect tear film instability and as a consequence, evaporative DED [3]. The former is supported by our observation that TFT and Schirmer were fairly correlated in patients with abnormal Schirmer values. This idea is also supported by a previous study that measured tear meniscus using OCT in a mixed group of DED patients [36]. In particular, the data shows that in patients with aqueous deficient DED, lower tear volume correlated well with severity of corneal epithelial damage. By contrast, higher tear volume correlated with corneal epithelial defects in patients with Meibomian gland dysfunction [36].

Several non-invasive techniques have been realized to measure tear dynamics, tear clearance and TFBUT non-invasively [1,37,38,39,40,41], but it is currently unknown how these measurements concur. In the present study, TFT was measured only for approximately 3 s after blinking using UH-OCT. We have, however, previously shown that evaporation rates can be measured from tear film en-face image serial recordings over several seconds obtained using high-resolution OCT [42]. This makes OCT an attractive approach to study both aspects of DED, aqueous deficiency and excess evaporation. Additionally, recent work has also indicated that OCT can be used to quantify the tear film lipid layer [43,44], but this approach requires further validation.

The present study has strengths and weaknesses that require further discussion: The major strengths of the current study include the relatively large sample size, the availability of OCT images from both eyes and the standardized protocol for OCT tear film imaging. A limitation of the study relates to the selection of the study population: As the current analysis is based on a pooled analysis of baseline data from several clinical trial, the sample does not represent a randomly selected cross-sectional population but rather DED patients who participated in different clinical trials. As such neither signs nor symptoms of DED were associated with known risk factors of the disease such as age and gender [45,46]. As the present study was not designed to study the risk factors for DED, but rather to study the agreement between signs and symptoms of DED this does not interfere with the conclusion of the study.

Another limitation relates to the absolute values of TFT. It is important to understand that the measured TFT values are not only dependent on the imaging system but also on the estimator used for thickness extraction from the images. Indeed different approaches have been proposed that will result in slightly different estimates of TFT [20,42,43,47]. For example, TFT depends on the lateral position on the ocular surface and thus differences in the definition of the averaging area will lead to slightly different values. This is very well known from other applications of OCT such as measurement of retinal nerve fiber layer thickness or retinal thickness. Absolute values as obtained with different commercial OCT platforms of retinal nerve fiber layer thickness [48,49] and retinal thickness [50,51] diverge considerably from each other. This is almost entirely related to the software solutions provided, because disagreement between values gets very small when third-party software is used on all images [51]. To account for these possible inconsistencies, in the current study only data from studies that used the same software were included. Finally, it needs to be considered that the tear film is a highly dynamic and continuously moving structure [52,53]. Recent data indicates that movements of the precorneal tear film may occur during visual fixation, which are not necessarily aligned with the movements of the cornea [52]. High-resolution en-face imaging of the tear film may in future allow to get more insight in this phenomenon.

In conclusion, our data show that TFT as measured with high resolution OCT is associated with symptoms of DED, although the correlation is not very strong. Pre-corneal TFT is also weakly correlated to TFBUT and Schirmer I. In patients with abnormal Schirmer I, the correlation is, however, more pronounced indicating that these signs rather reflect the aqueous-deficient component of DED. Given that OCT can also be used to estimate evaporation rates and quantify lipid layer thickness it may be an interesting approach in studying signs of DED.

## Figures and Tables

**Table 1:** Tear film thickness (TFT), break up time (TFBUT), and Schirmer I test in 225 patients with dry eye disease. Data were reported separately for right and left eyes (mean ± SD). Differences between right and left eyes were analyzed by paired t-tests.

Parameter	Right Eyes	Left Eyes	*p*-Value
TFT (µm)	4.2 ± 0.5	4.2 ± 0.5	0.57
TFBUT (seconds)	8.8 ± 3.5	8.5 ± 3.5	0.31
Schirmer I (mm)	15.8 ± 3.4	16.0 ± 4.4	0.48

**Table 2:** Slope of regression line, r- and *p*-values between tear film thickness (TFT) as well as break up time (TFBUT) and Schirmer I test separately shown for right and left eyes.

TFT						
	Right Eyes			Left Eyes		
	Regression Slope	r-Value	*p*-Value	Regression Slope	r-Value	*p*-Value
**TFBUT**						
All values	1.62	0.25	<0.001	1.92	0.29	<0.001
Normal values	0.54	0.17	0.12	1.00	0.27	0.02
Abnormal values	0.44	0.13	0.14	0.28	0.08	0.34
**Schirmer I**						
All values	2.30	0.36	<0.001	3.01	0.37	<0.001
Normal values	0.09	0.02	0.85	0.91	0.16	0.07
Abnormal values	1.43	0.54	<0.001	2.08	0.50	<0.001

## Supplementary Materials

The following are available online at https://www.mdpi.com/2077-0383/9/11/3791/s1, Figure S1: Typical example of an ultrahigh-resolution optical coherence tomography image showing a cross section through the cornea.
