# Supplementary material for: The Association between Tear Film Thickness as Measured with OCT and Symptoms and Signs of Dry Eye Disease: A Pooled Analysis of 6 Clinical Trials
Source: J Clin Med. 2020 Nov 23;9(11):3791. doi: 10.3390/jcm9113791 (PMC7700265; doi:10.3390/jcm9113791)
Supplement: Supplementary file 1 [file jcm-09-03791-s001.pdf]

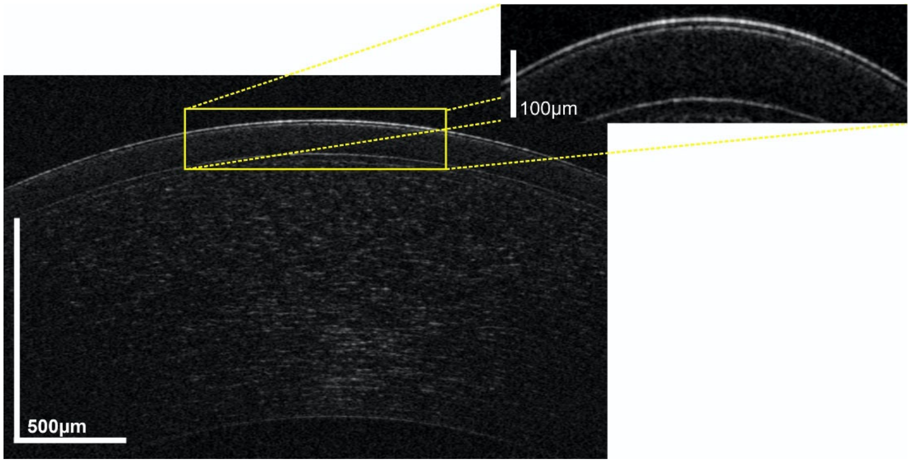

Supplement figure 1: Typical example of an ultrahigh-resolution optical coherence tomography image showing a cross section through the cornea. The yellow box depicts a magnified image of the corneal epithelial tissue and the precorneal tear film.
